# Interplay between the innate immune response and heart rate variability in healthy human volunteers

**DOI:** 10.1186/cc9678

**Published:** 2011-03-11

**Authors:** M Kox, BP Ramakers, JC Pompe, JG Van der Hoeven, CW Hoedemaekers, P Pickkers

**Affiliations:** 1 Radboud University Nijmegen Medical Centre, Nijmegen, the Netherlands

## Introduction

The autonomic nervous system (ANS) and innate immunity are intimately linked. Heart rate variability (HRV) analysis is a widely employed method to assess cardiac ANS activity, and changes in HRV indices may correlate with inflammatory markers. Here, we investigated whether baseline HRV predicts the innate immune response. Second, we investigated whether the magnitude of the inflammatory response correlated with HRV alterations.

## Methods

Forty healthy volunteers received a single intravenous bolus of 2 ng/kg endotoxin (lipopolysaccharide (LPS), derived from *Escherichia coli *O:113). Of these, 12 healthy volunteers were administered LPS again 2 weeks later. HRV was determined at baseline (just prior to LPS administration) and hourly thereafter until 8 hours post LPS. Plasma cytokine levels were determined at various time points.

## Results

**Figure 1:**
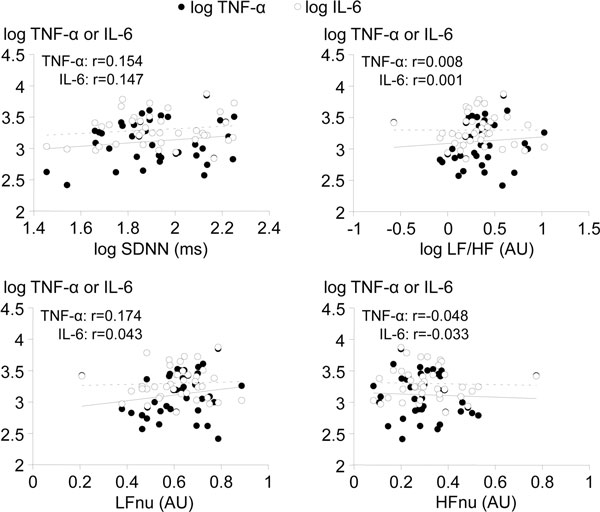
**Association between basal HRV indices (calculated at *t *= 0, just prior to LPS administration) and area under curve of the LPS-induced proinflammatory cytokine response (TNFα and IL-6, log pg/ml/hour) of 40 subjects**. ms, milliseconds; AU, arbitrary units. Solid and dashed lines, TNFα and IL-6 regression lines, respectively. Pearson correlation coefficients (none statistically significant) indicated.

Baseline HRV indices did not correlate with the magnitude of the LPS-induced inflammatory response. Despite large alterations in HRV following LPS administration, the extent of the inflammatory response did not correlate with the magnitude of HRV changes. In subjects that were administered LPS twice, inflammatory cytokines were markedly attenuated following the second LPS administration, while LPS-induced HRV alterations were similar. See Figure 1.

## Conclusions

HRV indices do not predict the innate immune response in a standardized model of systemic inflammation. The innate immune response results in HRV changes; however, no correlations with inflammatory cytokines were observed. These findings suggest that cardiac ANS activity may not reflect ANS outflow to other organs involved in the innate immune response. Furthermore, the magnitude of endotoxemia-related HRV changes does not reflect the extent of the inflammatory response.

